# An innovative single‐base extension method for synchronous detection of point mutations and MSI status in colorectal cancer

**DOI:** 10.1002/cam4.5557

**Published:** 2022-12-30

**Authors:** Li Liang, Xin Li, Lin Nong, Weijing Cai, Jixin Zhang, Ping Liu, Ting Li

**Affiliations:** ^1^ Peking University First Hospital Beijing China; ^2^ Shanghai Tongshu Biotechnology Co., Ltd Shanghai China

**Keywords:** colorectal cancer, MASE‐CE, MSI, single‐base extension, timesaver

## Abstract

**Background:**

An accurate genotyping analysis is one of the critical prerequisites for patients with colorectal cancer receiving matched therapies. Conventional genotyping analysis is currently used to detect either gene mutations or MSI status, delaying the detection of critical tumor biomarkers and thus the optimal time for treatment. An assay that analyzes both biomarkers in a streamlined process is eagerly needed.

**Methods:**

We developed an assay combining Multiplex PCR Amplification, Single‐base Extension and capillary electrophoresis (CE) analysis (MASE‐CE) for synchronous detection of *KRAS*/*NRAS*/*BRAF* mutations and MSI status. In a 190 colorectal cancer cohort, we identified seven somatic mutations in *KRAS*, *NRAS* and *BRAF* as well as five MSI loci (D2S123/D5S346/D17S250/BAT‐25/BAT‐26) simultaneously. *KRAS*/*NRAS*/*BRAF* mutations were detected by NGS and MASE‐CE, and MSI status were detected by PCR‐CE and MASE‐CE methods.

**Results:**

The MASE‐CE method showed high consistency with NGS for mutation detection (Kappa value ≥0.8) and PCR‐CE (Kappa value = 0.79). In addition, the limits of detection (LOD) of MASE‐CE assay for MSI and somatic mutation were 5% and 2%, respectively.

**Conclusions:**

In somatic mutation detection and MSI detection, the LOD of MASE‐CE assay was superior to that of qPCR and NGS. MASE‐CE assay is a highly sensitive, time‐saving and specimen‐saving method, which can greatly avoid the cumbersome testing process and provide clinical decision for doctors in time.

## Graphical abstract

An accurate genotyping analysis is one of the critical prerequisites for patients with colorectal cancer receiving matched therapies. We developed an assay combining multiplex amplification with single‐base extension assays for synchronous detection of KRAS/NRAS/BRAF mutations and MSI status. In somatic mutation detection and MSI detection, the LOD of MASE‐CE assay is superior to that of qPCR and NGS.

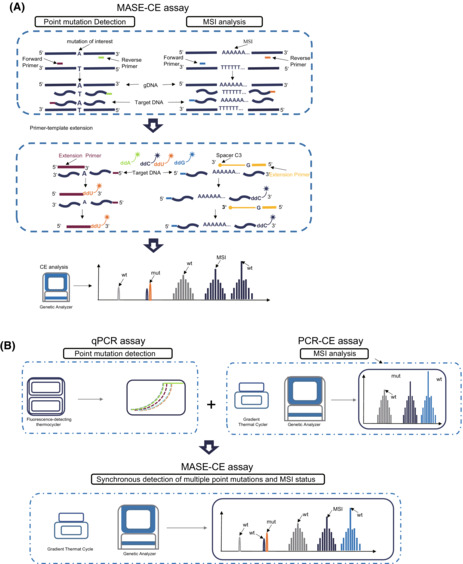

## 1 INTRODUCTION

The latest global cancer statistics reported that over 1.8 million new colorectal cancer (CRC) cases and 881,000 deaths were estimated to occur in 2020, accounting for about 1 in 10 cancer cases and deaths.^1^ More than 50% of cases will ultimately develop metastatic CRC (mCRC), with a high mortality rate and a five‐year survival rate of less than 10%.^2^ According to the National Comprehensive Cancer Network Clinical Practice Guidelines in Oncology (NCCN Guidelines for Colon Cancer/Rectal Cancer), molecular testing is essential for clinical management and treatment decision for CRC. Mutations in *KRAS*, *NRAS*, and *BRAF* constitute important biomarkers to guide clinical care of CRC. A review showed that there are currently no effective targeted therapies for 35%–40% of patients with KRAS or NRAS variants. Targeted combination therapy with a BRAF and EGFR inhibitor extended overall survival to 9.3 months in 5% to 10% of patients with a BRAF V600E sequence variant, compared with 5.9 months in patients receiving standard chemotherapy. For 5% of patients with microsatellite instability or mismatch repair deficiency, immunotherapy can be used in the first or subsequent lines, and the median overall survival of patients treated was 31.4 months.^3^ Advanced CRCs with *KRAS* (46.4%), *NRAS* (3.2%), or *BRAF* (3.5%) mutations portend a worse prognosis largely because of a lack of response to targeted agents and more aggressive biology.^4^, ^5^ Moreover, the microsatellite instability‐high (MSI‐H) phenotype is present in 15% of early stage mCRC, and MSI‐H has been shown to confer a good prognosis in patients with localized disease.^6^ Other evidence suggests that 5‐fluorouracil (5‐FU) and related drugs, such as capecitabine, actually worsen prognosis when given as a single agent to patients with early‐stage MSI‐H colorectal cancer.^7^ Therefore, it is imperative to develop rapid, accurate and reliable molecular detection technology to identification somatic mutations and MSI status.

The clinical benefit from biomarker‐targeted therapies and immune checkpoint therapies for patients with CRC relies on accurate genetic variant profiling. Currently, the standard methods for detecting *KRAS*, *NRAS*, and *BRAF* mutations include real‐time quantitative polymerase chain reaction (qPCR)‐based assay, amplification refractory mutation system (ARMS) assay and next generation sequencing (NGS) assay.^5^ For MSI detection, an increasing number of techniques have been well developed, especially PCR‐based methods.^2^, ^8^, ^9^, ^10^, ^11^ At present, PCR with fluorescent primers‐capillary electrophoresis (PCR‐CE) remains the gold standard for MSI detection. Recently, NGS has been also introduced to detect MSI, so that *KRAS*/*NRAS*/*BRAF* mutations and MSI status can be detected synchronously by NGS. However, NGS has a complicated detection process that is usually close to 10 days during routine clinical testing.

In our study, we have made a breakthrough in the application of MESE‐CE to achieve synchronous detection of somatic mutations and MSI status in CRC. We further applied a 190 colorectal cancer cohort of clinical samples to validate this method, and the results showed that MASE‐CE was an efficient, economical, time‐saving (8 hours vs. 7 days), and specimen‐saving (2 slices of FFPE tissue sample) method.

## 2 MATERIALS AND METHODS

### 2.1 Clinical sample collection

MASE‐CE is an innovative research in detection technology. To validate the performance of MASE‐CE, we retrospectively collected FFPE tissues from CRC patients undergoing surgery at Peking University First Hospital between October 2019 and February 2020, with no samples from neoadjuvant therapy procedures. The project was approved by the Ethics Committee of Peking University First Hospital. Patients signed informed consent and prospectively collected samples for exploratory biomarker studies in accordance with institutional review board guidelines. Excluding patients with cancer other than colorectal cancer, a total of 190 patients diagnosed with stage 0/IV CRC were enrolled (Table S1).

### 2.2 Study design

MASE‐CE assay was developed to detect seven mutations in KRAS (G12C/G12D/G13D/A146T), NRAS (G12C/G12D) and BRAF (V600E) as well as five MSI loci (D2S123/D5S346/D17S250/BAT‐25/BAT‐26) simultaneously. In a cohort of 190 colorectal cancer samples, MASE‐CE simultaneously detected seven mutation sites in *KRAS*, *NRAS*, and *BRAF*, as well as the status of five sites in MSI. At the same time, NGS and PCR‐CE were used as comparison methods to detect gene mutation sites and MSI status changes, respectively. A polymorphic pentanucleotide repeat (Penta C) was included as internal control to identify sample mix‐ups and contamination problems. Genomic position and sequencing information for all sites were obtained from NCBI's reference sequence (RefSeq) database. Primers for multiplex PCR amplification and single‐base extension were designed using Primer Premier 5.0 software. Since FFPE tissue tends to be highly fragmented, the amplicon length was restricted to a maximum of 300 nt, so that the amplification primers and the primary PCR products ranged from 25 to 300 nt. The single‐base extension primer for somatic mutation detection consisted of a segment binding up to the mutation site of interest and a tail with a specific length designed for separation, such as a 10 nt long 5′ anchor tail (5′‐ACGTTGGATG‐3′). For MSI detection, primers instead of DNA were extended. At the 3′‐OH of the MSI extension primer, an extension‐inhibiting compound C3 spacer was attached to avoid DNA extension. A complementary DNA sequence (at least 20 nt) was required for each extension primer. Extension primers were designed to be of different lengths to achieve separation by CE analysis.

### 2.3 Mase‐CE FOR *KRAS*/*NRAS*/*BRAF* mutations and MSI status

#### 2.3.1 DNA extraction

DNA was extracted from specimens using FFPE Nucleic Acid Extraction kit (FD‐50, Shanghai Tongshu Biotechnology Co.), and was eluted with 50 μl of Tris buffer (pH 7.5). Subsequently, DNA was quantified on Nanodrop 2000 (Thermo Fisher Scientific Inc).

#### 2.3.2 Multiplex PCR amplification

Different combinations of multiplex PCRs were performed to obtain robust amplifications for each amplicon. Multiplex PCR amplification was performed on an A200 Gradient Thermal Cycler (LongGene) in a volume of 20 μl containing 2 μl of DNA template and 18 μl of PCR solution (mixture of primer, Taq DNA polymerase, dNTP and Mg^2+^). Thermal Cycler conditions were: 50°C for 2 min, 1 cycle of 95°C for 2 min, 49 cycles of 95°C for 10 sec, 62°C for 15 sec, 72°C for 1 min and finally 5 min at 72°C. Multiplex PCR products were purified by adding 2 μl of the mixture of exonuclease I and shrimp alkaline phosphatase, followed by incubation at 37°C for 15 min, 80°C for 15 min and finally at 4°C.

#### 2.3.3 Single‐base extension

For mutation detection, the purified PCR products were used as templates, and unlabeled oligonucleotide primers were extended in the presence of the four fluorescently labeled dideoxynucleotides (ddNTPs), while for MSI detection, the extension primers were used as templates and the purified PCR products were extended. The extension reactions were performed on an A200 Gradient Thermal Cycler (LongGene) in a volume of 20 μl containing 9.5 μl of purified PCR products, 10 μl of single‐base extension solution (310,100, Shanghai Tongshu Biotechnology Co., Ltd) and 0.5 μl of primer mixture. Thermal Cycler conditions were: 1 cycle of 95°C for 2 min, 35 cycles of 95°C for 10 sec, 35 sec at 62°C, followed by rapid thermal ramp to 4°C. The unincorporated fluorescently labeled ddNTPs were removed by adding 3 μl of shrimp alkaline phosphatase solution to 17 μl of extension products, followed by incubation at 37°C for 60 min, 65°C for 15 min and finally at 4°C.

#### 2.3.4 Capillary electrophoresis (CE) analysis

1 μl of labeled extension products was mixed with 9 μl Hi‐Di™ Formamide (Thermo) and the products were denatured at 95°C for 5 min and 1°C for 10 min. Then, the products were separated using ABI Prism 3500 Dx Genetic Analyzer (Applied Biosystems). Data were analyzed using GeneMapper software (Applied Biosystems).

### 2.4 NGS for somatic mutation detection

NGS panel for 14 genes was performed on the Ion Torrent platform (Shanghai Tongshu Biotechnology Co., Ltd). The average sequencing depth was 15,000X with mapping rate > 95%.

### 2.5 PCR‐CE for MSI detection

PCR amplification was performed on an A200 Gradient Thermal Cycler (LongGene) in a volume of 10 μl containing 5 μl 2 × PCR Master Mix, 2 μl 5 × primer mix, 0.2 μl Amplitaq Gold DNA polymerase (5 units/μL), and 5 ng DNA templates. Thermal Cycler conditions were: 95°C for 4 min, 30 cycles of 95°C for 30 sec, 60°C for 30 sec, 72°C for 30 sec and 60°C for 45 min. PCR products were detected and analyzed by ABI 3730 genetic Analyzer (Applied Biosystems) following the manufacturer's protocol. Data analysis was performed using GeneScan Analysis and Genotyper Software packages (Applied Biosystems). MSI status was determined by the number of allelic bases and the internal control index.

### 2.6 Limits of detection and sensitivity of MASE‐CE

Furthermore, to assess the LOD and sensitivity of the MASE‐CE assay, mutant genes with different VAF were analyzed (Table S2). The wild type DNAs (293 T cell line) were purchased from the Cell Bank of Type Culture Collection of the Chinese Academy of Sciences. The mutant DNAs with alterations in seven SNP sites and five MSI loci (synthetic plasmids) were purchased from Sangon Biotech Co., Ltd. For each SNP site or MSI locus, four standards with different VAF were used (Table S3). To determine LOD of MASE‐CE, standard A1‐A7 was diluted 1.5 and two times with wild type DNA, and standard A8‐A12 was diluted 2.5 and four times.

### 2.7 Statistical methods

Kappa statistic was used to analyze the consistency of MASE‐CE and NGS for somatic mutation detection, and the consistency of by MASE‐CE and PCR‐CE for MSI detection. SPSS 25.0 (IBM Corp., Somers) was used.

## 3 RESULTS

### 3.1 MASE‐CE ASSAY

**FIGURE 1:**
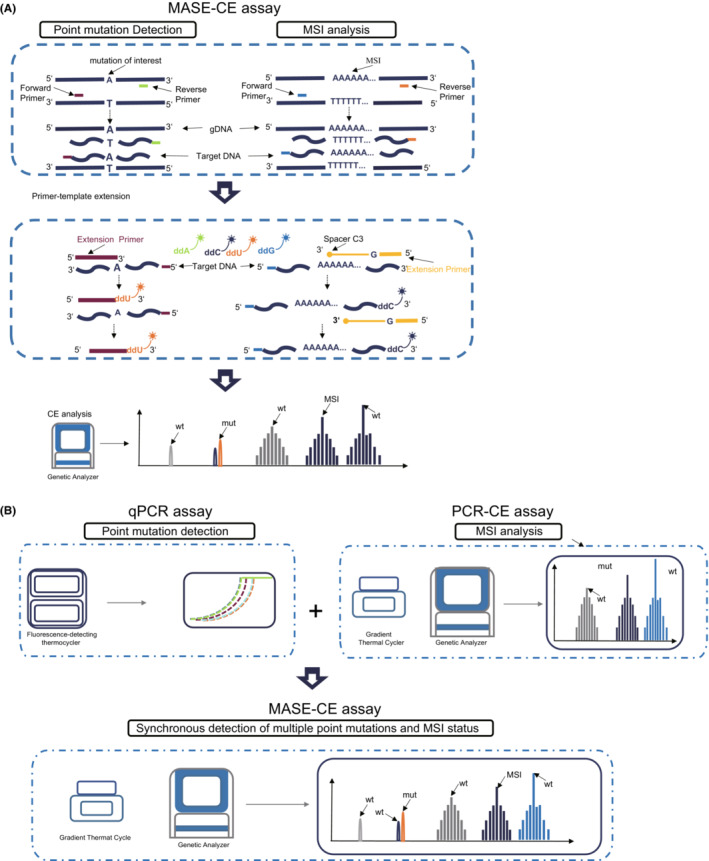
Comparation of MASE‐CE assay with Single Base Extension assay and PCR‐CE assay. (A) The principle of MASE‐CE assay for for synchronous detection of KRAS/NRAS/BRAF mutations and MSI status, which consists of three steps including multiplex PCR amplification, single‐base extension and CE analysis. (B) MASE‐CE assay detect somatic mutations and MSI status, which can be detected by qPCR and PCR‐CE, respectively. ddA, ddG, ddC and ddU represent different single labeled ddNTP (dideoxynucleotide). mut: mutant‐type; wt: wild‐type.

**FIGURE 2:**
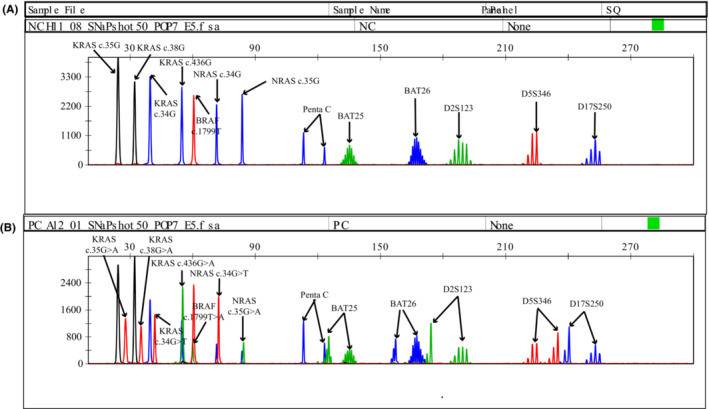
The signals of mutated genes and altered MSI locus by MASE‐CE assay. (A) wild type. (B) mutated genes and altered MSI locus. Each peak corresponds to a specific extended primer and extended template. Positions of nucleotides are indicated at the top of the figure. Lines indicate the location of each mutation and microsatellite. A = green; U = red; C = black; G = blue.

MASE‐CE assay consists of three steps including multiplex PCR amplification, single‐base extension and CE analysis. The unique extension primers used in the method were designed to be of different lengths to achieve separation by CE analysis (Figure 1A). The amplification product of the target region is used as a template for single‐base extension, and is carried out in a reaction system containing polymerase, four fluorescently labeled ddNTPs, and extension primers of different lengths. For the detection of gene point mutations, the primers added here are oligonucleotide sequences of different lengths that are complementary to the front of the site to be detected in the corresponding template. For the detection of MSI, the “primer” added here does not function as a primer in the reaction, but an oligonucleotide sequence complementary to the 3′ end of the corresponding purified product. The 3′ end of the primer is not extended due to C3‐Spacer blocking modification. On the contrary, the corresponding purified product is used as a primer here, and the oligonucleotide sequence is used as a template for single‐base extension. Through this process, one base is added on the basis of the originally set amplicon length of each site of MSI, so that fluorescently labeled bases of specific colors can be incorporated without changing the relative length of the amplicon of each site of MSI. Therefore, it is beneficial to measure the change in the size of the short repeats of the MSI site and to realize the combined detection with the point mutation of the gene. So that, MASE‐CE assay was developed to detect seven somatic mutations in KRAS (G12C/ G12D/ G13D/ A146T), NRAS (G12C/ G12D) and BRAF (V600E) as well as five MSI loci (D2S123/D5S346/D17S250/BAT‐25/BAT‐26) simultaneously. Figure 1B shows the schematic diagram of the instrument used for MASE‐CE detection, which is simplified on the basis of the original fluorescent quantitative PCR detection of point mutations and PCR‐CE detection of MSI, only a gradient thermal cycler and a genetic analyzer are required. As shown in Figure 2A,B, seven somatic mutations and five MSI loci were well separated on Genetic Analyzer.

### 3.2 LOD and sensitivity of MASE‐CE

**FIGURE 3:**
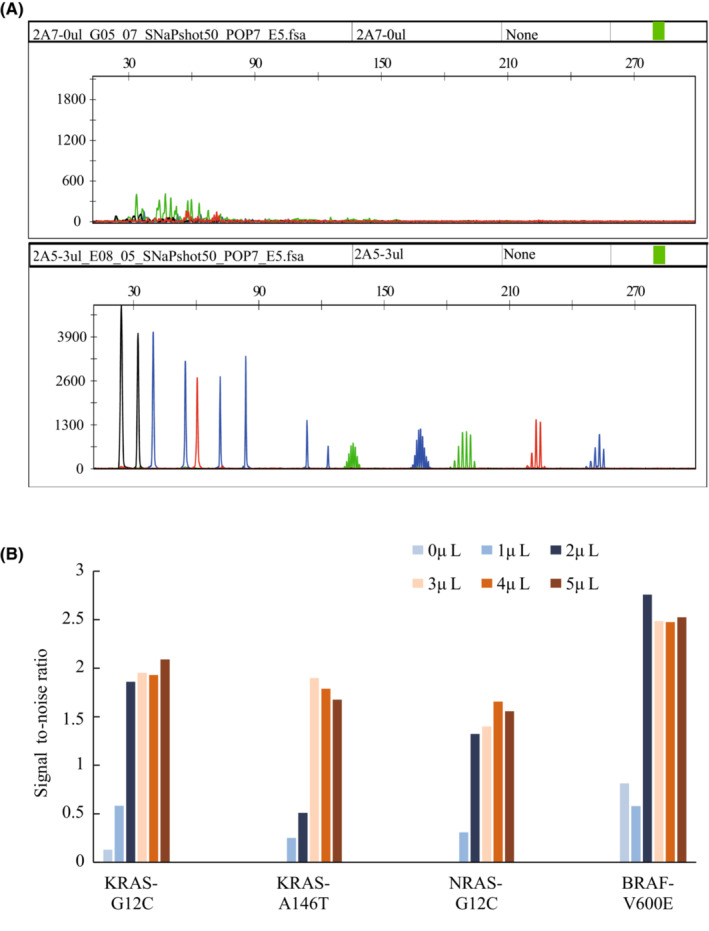
Effect of reaction mixture purification on the Signal‐to‐Noise ratio (SNR). (A) Results of somatic mutation and MSI status detection by MASE‐CE without purification (top panel) or with purification (bottom panel). (B) Different SNR with different amount of enzyme for purification. Signal‐to‐Noise ratio (SNR) equal to Expected peak/Extraneous peaks. KRAS‐G12C, KRAS c.34G > T; KRAS‐A146T, KRAS c.436G > A; NRAS‐G12C, NRAS c.34G > T; BRAF‐V600E, BRAF c.1799 T > A.

**FIGURE 4:**
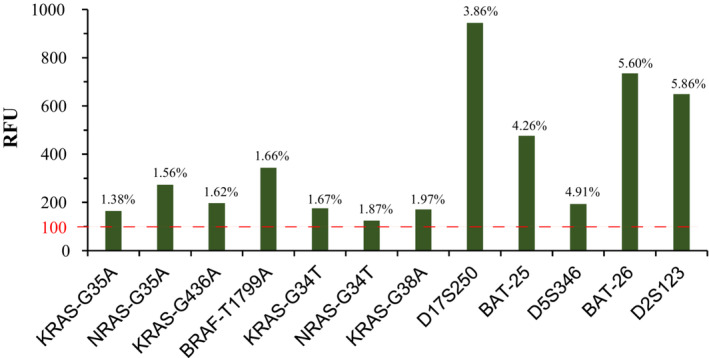
The LOD of several somatic mutations and MSI loci. KRAS‐G35A, KRAS^G12D^; NRAS‐G35A, NRAS^G12D^; KRAS‐G436A, KRAS^A146T^; BRAF‐T1799A, BRAF^V600E^; KRAS‐G34T, KRAS^G12C^; NRAS‐G34T, NRAS^G12C^; KRAS‐G38A, KRAS^G13D^.

We compared the results of MASE‐CE assay with or without purification of extension products. The results showed that the signal‐to‐noise ratio (SNR) with purification was higher than that without purification (Figure 3A), and the addition of 3 μl of shrimp alkaline phosphatase solution was optimal (Figure 3B). We assessed the LOD and sensitivity of MASE‐CE assay by diluting mutant DNAs to different VAF using wild type DNAs. MSI locus could be detected when mutant DNA accounted for <5% of the total input DNA, and somatic mutation of KRAS, NRAS and BRAF could be detected when mutant DNA accounted for <2% of the total input DNA (Figure 4). The detection sensitivity of MASE‐CE assay for MSI detection and somatic mutation detection were 5% and 2%, respectively (Table 1).

**TABLE 1:** Detection sensitivity assessment of MASE‐CE

	DNA Input (ng/uL)	Repeat 1	Repeat 2	Repeat 3	Average	Standard Deviation (SD)	CV (%)	Mutation frequency	Sensitivit (%)
KRAS‐G12C	10	181	270	129	193.33	58.21988969	30.11	0.0276	2.22
	5	140	115	212	176	41.12041937	23.36	0.0167	
KRAS‐G12D	10	224	229	213	222	6.683312552	3.01	0.0227	1.38
	5	176	170	245	164.67	34.02939905	20.67	0.0138	
KRAS‐G13D	10	355	301	228	294.67	52.04058245	17.66	0.0325	2.61
	5	169	142	202	171	24.53568829	14.35	0.0197	
KRAS‐A146T	10	170	159	247	192	39.14928692	20.39	0.0267	2.15
	5	216	140	235	197	41.04469109	20.83	0.0162	
NRAS‐G12C	10	138	123	128	129.67	6.236095645	4.81	0.0307	1.87
	5	99	166	109	124.67	29.51082664	23.67	0.0187	
NRAS‐G12D	10	255	250	283	262.67	14.52201394	5.52	0.0257	2.07
	5	259	300	260	273	19.09624745	6.99	0.0156	
BRAF‐V600E	10	225	217	441	294.33	103.7604078	35.23	0.0273	2.20
	5	289	271	471	343.67	90.33763827	26.29	0.0166	
MSI‐D2S123	10	879	895	1396	1056.67	240.0337939	22.72	0.1107	5.86
	5	559	773	615	649	90.61272905	13.96	0.0586	
MSI‐D5S346	10	321	276	380	325.67	42.58586098	13.08	0.0937	4.91
	5	183	174	221	192.67	20.36882149	10.57	0.0491	
MSI‐D17S250	10	1140	961	984	1028.33	79.51659505	7.73	0.0743	3.86
	5	737	1056	1039	944	146.5355475	15.52	0.0386	
MSI‐BAT25	10	769	874	582	741.67	120.7651532	16.28	0.0818	4.26
	5	396	644	387	475.67	119.0863366	25.04	0.0426	
MSI‐BAT26	10	1193	1141	1278	1204	56.46828018	4.69	0.106	5.6
	5	835	806	566	735.67	120.5551971	16.39	0.056	

Abbreviation: CV: Coefficient of Variance.

### 3.3 Clinical and pathological features of CRCS for MASE‐CE assay validation

The clinical and pathological features of 190 patients with CRC were summarized in Table S4. There were 73 females (38.4%) and 117 males (61.6%). Patients older than 60 years accounted for 62.6% (119/190), and the median age was 62 years (ranged from 35 to 86 years). There were 74 patients with rectal cancer (38.9%), 49 with colon cancer on the left side (25.8%), 58 with colon cancer on the right side (30.5%), and nine patients lack of pathogenic site information (4.7%). Patients with intermediate differentiation accounted for 71.1%, and 98.4% of patients were nonmucinous CRCs. As shown in Table S5, the mutation frequencies of KRAS, BRAF and NRAS in CRCs were about 31%, 1% and 0.5%, respectively. The consistency of somatic mutation detection by MASE‐CE and NGS was estimated, and the Kappa value was 0.95, 0.80 and 1.00 for detection of KRAS, BRAF and NRAS, respectively. Patients with MSI‐high accounted for about 6%. The consistency of MSI detection by MASE‐CE and MASS PCR‐CE was estimated, and the Kappa value was 0.794 (Table S6). The detection results of point mutation and MSI status of each patient and the consistency comparison between MASE‐CE and NGS and PCR‐CE methods were presented in Table S7.

### 3.4 Comparison of different methods to detect somatic mutations and MSI status

PCR‐based method is the commonly used method to detect somatic mutations or MSI. Detection of somatic mutation and MSI was performed using qPCR and PCR‐CE (Figure 1D), respectively. It took almost 5 hours for detecting somatic mutations and MSI respectively.^2^, ^12^ It requires 50–100 ng of input DNA (5–10 slices of FFPE sample) for qPCR and 5–10 ng of input DNA (2 slices of FFPE sample) for PCR‐CE.^12^, ^13^ NGS can be used to detect somatic mutations and MSI simultaneously. It takes at least1‐2 weeks and requires 50–250 ng of input DNA (10–15 slices of FFPE sample).^9^ The newly developed method, MASE‐CE, has the ability to detect somatic mutations and MSI simultaneously, and it only takes 8 hours and requires 30 ng of input DNA (2 slices of FFPE sample). The LOD for somatic mutation detection was 1%–5%, 3% and 2% by qPCR, NGS and MASE‐CE, respectively; and the LOD for MSI detection was 2%–10%, 20% and 5% by PCR‐CE, NGS and MASE‐CE, respectively. MASE‐CE has the lowest cost for a single detection of both somatic mutations and MSI. The comparison of MASE‐CE, NGS, qPCR and PCR‐CE was summarized in Table 2.

**TABLE 2:** Comparison of technical advantages between MASE‐CE and NGS and CE assay

Detection target	MASE‐CE	NGS	qPCR	PCR‐CE
	Point mutation; MSI status	Point mutation; MSI status	Point mutation	MSI status
Detection period	8 h	1–2 weeks	5 h	5 h
DNA input	30 ng	50–250 ng	50–100 ng	5–10 ng
Limit of Detection (LOD)				
Somatic mutation	2%	3%	1–5%	\
MSI status	5%	20%	\	2–10%
FFPE thin‐sliced sections	2	10–15	5–10	2
Economic cost	low	high	medium	medium

## 4 DISCUSSION

In this study, on the basis of the standard pipeline of single‐base extension assay for mutation detection,^14^, ^15^ we implanted two procedures to detect MSI: PCR amplification of MSI loci and fluorescently labeling of MSI loci. To fluorescently label MSI loci using single‐base extension that has been applied to label mutation sites, we designed the extension primer for MSI detection as the template and the DNAs of MSI loci were extended using fluorescently labeled ddNTPs. Therefore, MSI loci and mutation sites can be fluorescently labeled simultaneously and fragment analysis can be conducted to detect fluorescently labeled MSI loci and mutation sites simultaneously. To our knowledge, this is the only method that can achieve synchronous detection of mutation and MSI at one time, other than NGS. We assessed the LOD of MASE‐CE assay by diluting mutant DNAs. Compared with NGS for MSI detection, the LOD of MASE‐CE (5%) was much lower than that of NGS (20%) (Table 2). And compared with qPCR, for detecting *KRAS*, *NRAS*, and *BRAF* mutations, MASE‐CE had comparable performance with <2% LOD.^16^ Therefore, the MASE‐CE assay is sufficient for clinical tissue samples.

RAS mutation is the most frequent oncogenic alteration in human cancers, and KRAS is the most frequently mutated followed by NRAS. The emblematic KRAS mutant cancers are pancreatic (90%),^17^, ^18^ colorectal (42.6%),^19^, ^20^ lung adenocarcinomas (27%)^21^ and urogenital cancers.^22^ KRAS mutations in CRCs affect codon 12 (>75%), 13, and 61, and CRCs also have NRAS mutations (<5%) at codon 12, 13, or 61.^19^ The gene for *BRAF*, was mutated in approximately 5–10% of CRCs. Recent researches have shown that missing values for some genomic markers such as KRAS, BRAF^V600E^ mutations and MSI status were in the range of 30%–50% in some cohorts.^23^ Activating mutations in NRAS account for 20%–30% of melanoma.^24^ Therefore, the MASE‐CE assay for detecting KRAS, NRAS, BRAF mutations and MSI status can also be applied to patients with other cancers, including pancreatic and lung adenocarcinomas.

To validate the performance of the MASE‐CE assay for detecting *KRAS*, *NRAS*, *BRAF* mutations and MSI status on clinical samples, we assessed the consistency of MASE‐CE and NGS for mutation detection or PCR‐CE for MSI detection in 190 patients with CRC. The kappa value was 0.95, 0.80 and 1.00 for detecting mutation in *KRAS*, *BRAF* and *NRAS*, respectively (Table S5). And the kappa value was 0.794 for detecting MSI (Table S6). In our study, the MSI status results of 3 cases (1.6%) by MASE‐CE were inconsistent with that by PCR‐CE assay. The results showed that MASE‐CE was a reliable method in clinical use. We compared MASE‐CE with clinically widely used approaches. Compared with PCR‐CE, the sensitivity, specificity, positive predictive value (PPV) and negative predictive value (NPV) of MASE‐CE for MSI detection was 91.7%, 98.3%, 78.6% and 99.4%, respectively (Table S8). Compared with NGS, the sensitivity, specificity, PPV and NPV of MASE‐CE for mutation detection was 95.4%, 100%, 100% and 97.6%, respectively (Table S9). The results showed that MASE‐CE had high consistency with general methods, indicating that MASE‐CE was a reliable method. Moreover, MASE‐CE workflow from DNA extraction to results just took 8 hours and required lower DNA input (30 ng versus 50–250 ng) when compared to NGS assay. In addition, MASE‐CE also had comparable performance to PCR‐CE, the gold standard method for MSI detection, in terms of time consumption, sample input and LOD (Table 2). Therefore, MASE‐CE assay is a time‐saving (8 hours versus 7 days) and specimen‐ saving (2 slices versus 10–15 slices of FFPE tissue sample) method.

Nevertheless, there were several limitations of MASE‐CE in clinical practice. First, only frequently altered mutation sites were included (KRAS^G12C^, KRAS^G12D^, KRAS^G13D^, KRAS^A146T^, NRAS^G12C^, NRAS^G12D^ and BRAF^V600E^), which may omit information about mutations at other sites. Given the ever‐increasing demand for clinical tumor genotyping, a high throughput sequencing was urgently needed and the MASE‐CE assay could be further improved by adding emerging biomarker that were especially informative in CRC. Second, due to the trace amount of circulating tumor DNA in blood samples (<1%), MASE‐CE assay cannot be applied to test blood samples. An optimized reaction/enzyme system, in combination with the design of primers, optimization of the reaction parameters (including primer selection, PCR reaction system and program optimization), will be a new breakthrough in improving the performance of MASE‐CE assay in the future. Third, the LOD of NGS for MSI testing and the MASE‐CE test for validation in other cancer patients have not yet been evaluated and will be assessed in the following work.

Conventional detection techniques, such as SNaPshot Multiplex Assay^25^ and qPCR,^2^ could be used to detect somatic mutation rather than MSI status. Currently, the most common MSI status detection technologies, including NGS^9^ and PCR‐CE,^12^ were not only time‐consuming, but also low sensitivity, which cannot meet the clinical practice. Additionally, NGS is an expensive method with a lack of availability in most parts of the world. MASE‐CE assay uses a unique single‐base extension method, which completely breaks the current detection technology that can only detect multiple point mutations or MSI status. The performance of the MASE‐CE assay further shows that it has high sensitivity and low LOD for clinical practice.

## 5 CONCLUSIONS

In general, we constructed a novel method, MASE‐CE, which can synchronous detect *KRAS*, *NRAS*, *BRAF* mutations and MSI status of CRC. Compared with the traditional clinical diagnostic methods, it has the advantages of high efficiency, high sensitivity, simple operation, time‐saving and specimen‐saving (Figure 1B and Table 2).

## AUTHOR CONTRIBUTIONS

All authors confirmed they have contributed to the intellectual content of this paper and have met the following 4 requirements: (a) significant contributions to the conception and design, acquisition of data, or analysis and interpretation of data; (b) drafting or revising the article for intellectual content; (c) final approval of the published article; and (d) agreement to be accountable for all aspects of the article thus ensuring that questions related to the accuracy or integrity of any part of the article are appropriately investigated and resolved. L. Liang., X. Li., and J. Zhang. performed the experimented and analyzed the data. P. Liu. and L. Nong. performed the experiments, analyzed the data, and wrote the manuscript. W. Cai. supervised aspects of the project. T. Li. conceived and managed the project.

## FUNDING INFORMATION

Roche Diagnostics.

## CONFLICT OF INTEREST

Upon manuscript submission, all authors completed the author disclosure form. Disclosures and/or potential conflicts of interest:

## ETHICAL APPROVAL

The study was approved by the ethics committee of Peking University First Hospital. Written informed consent was obtained from all individuals included in the study.

## CONSENT FOR PUBLICATION

All authors confirmed they have contributed to the intellectual content of this paper and have met the following 4 requirements: (a) significant contributions to the conception and design, acquisition of data, or analysis and interpretation of data; (b) drafting or revising the article for intellectual content; (c) final approval of the published article; and (d) agreement to be accountable for all aspects of the article thus ensuring that questions related to the accuracy or integrity of any part of the article are appropriately investigated and resolved.

## ROLE OF SPONSOR

The funding organizations played no role in the design of study, choice of enrolled patients, review and interpretation of data, preparation of manuscript, or final approval of manuscript.

## Supporting information


Table S1.

Table S2.

Table S3.

Table S4.

Table S5.

Table S6.

Table S7.

Table S8.

Table S9.

## Data Availability

All data needed to evaluate the conclusions in the paper are present in the paper or the Supplementary Materials.
