# Supplementary material for: An innovative single‐base extension method for synchronous detection of point mutations and MSI status in colorectal cancer
Source: Cancer Med. 2022 Dec 30;12(7):8367–77. doi: 10.1002/cam4.5557 (PMC10134345; doi:10.1002/cam4.5557)
Supplement: Supplementary file 1 — Table S1. [file CAM4-12-8367-s001.doc]

**Supplementary Table 1** Clinical characteristics of patients.

| Patient | Gender | Age | TNM | Location |
| --- | --- | --- | --- | --- |
|
| P1 | Female | 56 | T3N2M0 | Rectal cancer |
| P2 | Female | 52 | T2N0M0 | Rectal cancer |
| P3 | Female | 77 | T3N0M0 | Other |
| P4 | Female | 68 | TisN0M0 | Rectal cancer |
| P5 | Female | 77 | T3N0M0 | Other |
| P6 | Male | 44 | T3N1bM0 | Other |
| P7 | Male | 58 | T3N0M0 | Rectal cancer |
| P8 | Female | 60 | T3N1bM0 | Rectal cancer |
| P9 | Female | 61 | T1N0M0 | Other |
| P10 | Male | 62 | T1N0M0 | Other |
| P11 | Female | 78 | T4N0M0 | Other |
| P12 | Male | 80 | T3N0M0 | Rectal cancer |
| P13 | Male | 56 | T3N1bM0 | Rectal cancer |
| P14 | Female | 84 | T3N0M0 | Other |
| P15 | Male | 63 | T2N0M1a | Other |
| P16 | Male | 49 | T3N0M0 | Right colon |
| P17 | Male | 72 | T2N0M0 | Left colon |
| P18 | Male | 56 | T3N2aM0 | Rectal cancer |
| P19 | Male | 76 | T3N0M0 | Rectal cancer |
| P20 | Male | 57 | T3N1aM1a | Other |
| P21 | Female | 61 | T2N0M0 | Other |
| P22 | Female | 70 | T3N0M1a | Other |
| P23 | Male | 66 | T3N1bM0 | Other |
| P24 | Female | 63 | T3N2aM0 | Other |
| P25 | Male | 35 | T3N1aM0 | Other |
| P26 | Male | 53 | T3N0M0 | Other |
| P27 | Male | 84 | T3N1aM0 | Rectal cancer |
| P28 | Female | 61 | T3N0M0 | Rectal cancer |
| P29 | Male | 56 | T3N0M0 | Other |
| P30 | Male | 75 | T3N0M0 | Rectal cancer |
| P31 | Female | 65 | T3N1aM0 | Other |
| P32 | Male | 54 | T3N1bM0 | Rectal cancer |
| P33 | Female | 71 | T3N1bM0 | Other |
| P34 | Male | 48 | T2N0M0 | Other |
| P35 | Male | 66 | T2N0M0 | Rectal cancer |
| P36 | Male | 66 | T1N0M0 | Other |
| P37 | Male | 47 | T4N1bM1b | Other |
| P38 | Female | 71 | T3N0M0 | Other |
| P39 | Male | 51 | T3N1bM0 | Rectal cancer |
| P40 | Female | 70 | T3N1bM0 | Other |
| P41 | Female | 85 | T3N0M0 | Other |
| P42 | Male | 56 | T2N0M0 | Other |
| P43 | Female | 64 | T2N0M0 | Other |
| P44 | Female | 63 | T1N0M0 | Other |
| P45 | Male | 50 | / | Other |
| P46 | Male | 60 | T2N0M0 | Other |
| P47 | Female | 55 | T3N0M0 | Other |
| P48 | Female | 59 | T2N0M0 | Other |
| P49 | Female | 80 | T3N2bM1b | Other |
| P50 | Female | 83 | T3N0M0 | Rectal cancer |
| P51 | Male | 64 | TXNXM0 | Other |
| P52 | Female | 63 | T4N1cM0 | Other |
| P53 | Male | 83 | / | Other |
| P54 | Male | 80 | T3N1bM0 | Other |
| P55 | Female | 50 | T3N2aM0 | Other |
| P56 | Male | 71 | T4N0M0 | Other |
| P57 | Female | 72 | T3N0M0 | Other |
| P58 | Female | 70 | T2N0M0 | Other |
| P59 | Female | 62 | T3N2aM0 | Other |
| P60 | Male | 41 | T3N0M1a | Other |
| P61 | Female | 45 | T3N0M0 | Other |
| P62 | Male | 61 | T3N0M0 | Other |
| P63 | Female | 79 | T3N1bM0 | Other |
| P64 | Male | 85 | T3N0M0 | Other |
| P65 | Male | 53 | T3N1aM0 | Rectal cancer |
| P66 | Female | 61 | T1N1aM0 | Rectal cancer |
| P67 | Male | 64 | T4N1aM1a | Other |
| P68 | Female | 47 | T3N0M0 | Other |
| P69 | Male | 40 | T4NxM1b | Other |
| P70 | Male | 55 | T3N0M0 | Other |
| P71 | Female | 70 | T1N0M0 | Other |
| P72 | Male | 84 | T3N0M0 | Other |
| P73 | Male | 49 | T3N1aM0 | Other |
| P74 | Female | 40 | T3N0M0 | Other |
| P75 | Female | 49 | T3N0M0 | Other |
| P76 | Male | 62 | T3N1bM0 | Other |
| P77 | Male | 73 | T3N0M0 | Other |
| P78 | Female | 84 | T3N0M0 | Other |
| P79 | Male | 75 | T3N1bM0 | Other |
| P80 | Male | 67 | T3N2bM1a | Other |
| P81 | Male | 52 | T3N0M0 | Other |
| P82 | Male | 81 | TxNxM0 | Other |
| P83 | Female | 46 | T3N0M0 | Other |
| P84 | Female | 44 | T3N0M0 | Other |
| P85 | Male | 66 | T2N0M0 | Other |
| P86 | Female | 66 | T2N1bM0 | Other |
| P87 | Male | 64 | T2N0M0 | Other |
| P88 | Male | 75 | T2N1aM0 | Other |
| P89 | Male | 69 | TisN0M0 | Other |
| P90 | Male | 66 | T4N1bM0 | Other |
| P91 | Female | 70 | T1N0M0 | Other |
| P92 | Male | 83 | T3N0M0 | Other |
| P93 | Male | 75 | T3N0M0 | Other |
| P94 | Male | 68 | T3N0M0 | Other |
| P95 | Female | 61 | T2N0M0 | Other |
| P96 | Female | 42 | T3N2bM0 | Other |
| P97 | Male | 56 | T4N1M1 | Other |
| P98 | Female | 53 | T1N0M0 | Other |
| P99 | Male | 62 | T3N0M0 | Other |
| P100 | Female | 61 | T4N2aM1a | Rectal cancer |
| P101 | Male | 74 | T3N1bM1a | Other |
| P102 | Male | 54 | T4N2bM0 | Other |
| P103 | Female | 60 | T4N1bM0 | Other |
| P104 | Male | 68 | T3N0M0 | Other |
| P105 | Male | 50 | T3N0M0 | Other |
| P106 | Male | 86 | T3N0M0 | Other |
| P107 | Female | 66 | T3N0M0 | Other |
| P108 | Male | 85 | T3N0M0 | Other |
| P109 | Female | 63 | T3N0M0 | Other |
| P110 | Male | 80 | T3N0M0 | Other |
| P111 | Female | 76 | T3N0M0 | Other |
| P112 | Male | 72 | T4N2aM1a | Other |
| P113 | Female | 57 | T3N0M0 | Other |
| P114 | Male | 80 | T3N1aM0 | Rectal cancer |
| P115 | Male | 67 | T3N0M0 | Other |
| P116 | Male | 52 | T3N2aM0 | Rectal cancer |
| P117 | Male | 73 | T3N1bM0 | Other |
| P118 | Male | 67 | T3N0M0 | Other |
| P119 | Female | 81 | T3N0M0 | Other |
| P120 | Female | 77 | T3N0M0 | Other |
| P121 | Female | 66 | TXNXMx | Other |
| P122 | Female | 73 | T4N1cM0 | Other |
| P123 | Male | 68 | T3N0M0 | Other |
| P124 | Female | 74 | T3N0M0 | Other |
| P125 | Male | 70 | T3N1bM0 | Other |
| P126 | Male | 71 | T3N2aM0 | Other |
| P127 | Male | 70 | T3N0M0 | Other |
| P128 | Male | 48 | T3N0M0 | Other |
| P129 | Male | 62 | / | Other |
| P130 | Male | 35 | T3N0M0 | Other |
| P131 | Male | 66 | T2N0M0 | Other |
| P132 | Male | 46 | T3N0M0 | Other |
| P133 | Female | 54 | T3N1cM0 | Other |
| P134 | Male | 55 | T2N1aM0 | Rectal cancer |
| P135 | Female | 60 | TXNXM1b | Other |
| P136 | Male | 39 | T1N0M0 | Other |
| P137 | Male | 68 | T4N1aM0 | Other |
| P138 | Female | 71 | T3N0M0 | Other |
| P139 | Male | 69 | T3N0M0 | Other |
| P140 | Female | 47 | T3N0M0 | Other |
| P141 | Male | 59 | T1N0M0 | Other |
| P142 | Male | 58 | T3N1aM0 | Other |
| P143 | Female | 49 | T3N1bM0 | Other |
| P144 | Male | 62 | T3N0M0 | Other |
| P145 | Male | 52 | T3N0M0 | Other |
| P146 | Male | 71 | T3N1aM1a | Other |
| P147 | Male | 40 | T4N0M0 | Other |
| P148 | Female | 53 | T3N2M1 | Other |
| P149 | Male | 46 | T3N0M0 | Other |
| P150 | Female | 60 | T3N0M0 | Other |
| P151 | Male | 49 | T3N1cM0 | Other |
| P152 | Male | 52 | T2N0M0 | Rectal cancer |
| P153 | Male | 55 | T3N1aM0 | Other |
| P154 | Male | 59 | T3N2bM0 | Other |
| P155 | Male | 76 | TXNXM1a | Other |
| P156 | Male | 58 | TXNXM0 | Other |
| P157 | Male | 56 | T3N0M0 | Other |
| P158 | Male | 60 | T3N0M0 | Other |
| P159 | Male | 68 | T1N0M0 | Other |
| P160 | Male | 74 | T2N0M0 | Other |
| P161 | Male | 56 | T3N0M0 | Other |
| P162 | Female | 43 | T4N2bM0 | Other |
| P163 | Female | 65 | T3N0M0 | Other |
| P164 | Male | 45 | Ti s N0M0 | Other |
| P165 | Male | 67 | T3N1bM0 | Other |
| P166 | Male | 60 | T4N2bM0 | Other |
| P167 | Male | 67 | T4N2bM0 | Other |
| P168 | Male | 60 | TXNXMX | Rectal cancer |
| P169 | Male | 53 | T1N0M0 | Other |
| P170 | Male | 62 | T1N0M0 | Other |
| P171 | Male | 73 | T2N0M0 | Other |
| P172 | Male | 26 | T3N1bM0 | Other |
| P173 | Male | 60 | T2N1bM0 | Other |
| P174 | Female | 49 | T3N1aM0 | Other |
| P175 | Female | 65 | T3N0M0 | Other |
| P176 | Male | 74 | / | Other |
| P177 | Female | 33 | T3N2aM0 | Other |
| P178 | Female | 59 | T3NXM1a | Other |
| P179 | Female | 59 | T3N0M0 | Other |
| P180 | Male | 66 | T3N0M0 | Other |
| P181 | Male | 73 | T3N1aM0 | Other |
| P182 | Female | 71 | T3N2bM0 | Rectal cancer |
| P183 | Female | 60 | T3N1bM1a | Other |
| P184 | Female | 55 | T3N0M1a | Other |
| P185 | Female | 52 | T2N0M0 | Other |
| P186 | Male | 59 | T3N1aM0 | Other |
| P187 | Male | 66 | T3N0M0 | Other |
| P188 | Male | 70 | T3N2aM1a | Other |
| P189 | Male | 60 | T3NXM1a | Other |
| P190 | Male | 61 | T4aN1bM0 | Other |
