# Supplementary material for: An innovative single‐base extension method for synchronous detection of point mutations and MSI status in colorectal cancer
Source: Cancer Med. 2022 Dec 30;12(7):8367–77. doi: 10.1002/cam4.5557 (PMC10134345; doi:10.1002/cam4.5557)
Supplement: Supplementary file 2 — Table S2. [file CAM4-12-8367-s009.doc]

**Supplementary Table 2** Mutant DNAs with different variant allele frequencies (VAFs) for LOD and sensitivity determination.

| Detection sites | VAF |
| --- | --- |
| KRAS-G34T | 4.08% |
|  | 2.76% |
|  | 1.67% |
| KRAS-G35A | 3.37% |
|  | 2.27% |
|  | 1.38% |
| KRAS-G38A | 4.79% |
|  | 3.25% |
|  | 1.97% |
| KRAS-G436A | 3.95% |
|  | 2.67% |
|  | 1.62% |
| NRAS-G34T | 4.54% |
|  | 3.07% |
|  | 1.87% |
| NRAS-G35A | 3.81% |
|  | 2.57% |
|  | 1.56% |
| BRAF-T1799A | 4.04% |
|  | 2.73% |
|  | 1.66% |
| D2S123 | 19.93% |
|  | 11.07% |
|  | 5.86% |
| D5S346 | 17.13% |
|  | 9.37% |
|  | 4.91% |
| D17S250 | 13.84% |
|  | 7.43% |
|  | 3.86% |
| BAT-25 | 15.12% |
|  | 8.18% |
|  | 4.26% |
| BAT-26 | 19.17% |
|  | 10.6% |
|  | 5.6% |
