# Supplementary material for: An innovative single‐base extension method for synchronous detection of point mutations and MSI status in colorectal cancer
Source: Cancer Med. 2022 Dec 30;12(7):8367–77. doi: 10.1002/cam4.5557 (PMC10134345; doi:10.1002/cam4.5557)
Supplement: Supplementary file 3 — Table S3. [file CAM4-12-8367-s007.doc]

**Supplementary Table 3** Standards of mutant DNAs with alterations in 7 SNP sites and 5 MSI loci.

| **Detection sites** | **Standards** | **VAF (%)** |
| --- | --- | --- |
| KRAS G12C | P1 | 71.93 |
|  | P13 | 38.44 |
|  | P25 | 6.67 |
|  | A1 | 4.08 |
| KRAS G12D | P2 | 42.83 |
|  | P14 | 25.64 |
|  | P26 | 6.19 |
|  | A2 | 3.37 |
| KRAS G13D | P3 | 73.78 |
|  | P15 | 33.64 |
|  | P27 | 8.08 |
|  | A3 | 4.79 |
| KRAS A146T | P4 | 67.69 |
|  | P16 | 38.89 |
|  | P28 | 7.43 |
|  | A4 | 3.95 |
| NRAS G12C | P5 | 57.56 |
|  | P17 | 31.63 |
|  | P29 | 7.78 |
|  | A5 | 4.54 |
| NRAS G12D | P6 | 57.09 |
|  | P18 | 38.70 |
|  | P30 | 7.45 |
|  | A6 | 3.81 |
| BRAF V600E | P7 | 61.23 |
|  | P19 | 39.82 |
|  | P31 | 7.92 |
|  | A7 | 4.04 |
| MSI-D2S123 | P8 | 55.45 |
|  | P20 | 33.24 |
|  | P32 | 29.32 |
|  | A8 | 19.93 |
| MSI-D5S346 | P9 | 50.82 |
|  | P21 | 34.07 |
|  | P33 | 20.53 |
|  | A9 | 17.13 |
| MSI-D17S250 | P10 | 57.23 |
|  | P22 | 41.26 |
|  | P34 | 21.94 |
|  | A10 | 13.84 |
| MSI-BAT25 | P11 | 56.50 |
|  | P23 | 43.80 |
|  | P35 | 23.77 |
|  | A11 | 15.12 |
| MSI-BAT26 | P12 | 63.36 |
|  | P24 | 45.36 |
|  | P36 | 29.33 |
|  | A12 | 19.17 |
| None | N1 | 0 |
