# Supplementary material for: An innovative single‐base extension method for synchronous detection of point mutations and MSI status in colorectal cancer
Source: Cancer Med. 2022 Dec 30;12(7):8367–77. doi: 10.1002/cam4.5557 (PMC10134345; doi:10.1002/cam4.5557)
Supplement: Supplementary file 4 — Table S4. [file CAM4-12-8367-s004.doc]

**Supplementary Table 4.** The clinical and pathological features of 190 patients with CRC.

| **Characteristic** | **No. of Cases (Proportion)** |
| --- | --- |
| Gender |  |
| Male | 117 (61.6) |
| Female | 73 (38.4) |
| Age |  |
| ≥60 years | 119 (62.6) |
| <60 years | 71 (37.4) |
| Location |  |
| Rectal cancer | 76 (40.0) |
| Left Colon | 54 (28.4) |
| Right Colon | 60 (31.6) |
| Clinical stage |  |
| 0 | 3 |
| I | 30 |
| IIA | 67 |
| IIB | 3 |
| IIIA | 5 |
| IIIB | 45 |
| IIIC | 7 |
| IVA | 17 |
| IVB | 4 |
| unknown | 9 |
| Differentiation |  |
| High | 8 (4.2) |
| intermediate | 135 (71.1) |
| Low | 16 (8.4) |
| Unknown | 31 (16.3) |
| Histological type |  |
| Mucinous | 3 (1.6) |
| Nonmucinous | 187 (98.4) |
