# Supplementary material for: An innovative single‐base extension method for synchronous detection of point mutations and MSI status in colorectal cancer
Source: Cancer Med. 2022 Dec 30;12(7):8367–77. doi: 10.1002/cam4.5557 (PMC10134345; doi:10.1002/cam4.5557)
Supplement: Supplementary file 5 — Table S5. [file CAM4-12-8367-s002.doc]

**Supplementary Table 5** The consistency of MASE-CE and NGS for detecting KRAS/NRAS/BRAF mutations.

|  |  |  | MASE-CE | |  | Kappa Value |
| --- | --- | --- | --- | --- | --- | --- |
|  |  |  | Positive | Negative | Total |  |
| NGS | KRAS | Positive | 58 | 3 | 61 | 0.951288296 |
|  |  | Negative | 1 | 128 | 129 |  |
|  |  | Ttotal | 59 | 131 | 190 |  |
|  | NRAS | Positive | 1 | 0 | 1 | 1 |
|  |  | Negative | 0 | 189 | 189 |  |
|  |  | Total | 1 | 189 | 190 |  |
|  | BRAF | Positive | 2 | 1 | 3 | 0.797441365 |
|  |  | Negative | 0 | 187 | 187 |  |
|  |  | Total | 2 | 188 | 190 |  |
