# Supplementary material for: An innovative single‐base extension method for synchronous detection of point mutations and MSI status in colorectal cancer
Source: Cancer Med. 2022 Dec 30;12(7):8367–77. doi: 10.1002/cam4.5557 (PMC10134345; doi:10.1002/cam4.5557)
Supplement: Supplementary file 6 — Table S6. [file CAM4-12-8367-s005.doc]

**Supplementary Table 6** The consistency of MASE-CE and PCR-CE for detecting MSI status.

|  |  | MASE-CE | | | Kappa Value |
| --- | --- | --- | --- | --- | --- |
|  |  | MSS/MSI-L | MSI-H | Total |  |
| PCR-CE | MSS/MSI-L | 175 (92.11%) | 3 (1.58%) | 178 (93.68%) | 0.794 |
|  | MSI-H | 1 (0.53%) | 11 (5.79%) | 12 (6.32%) |  |
|  | Total | 176 (92.64%) | 14 (7.37%) | 190 (100.00%) |  |
