# Supplementary material for: An innovative single‐base extension method for synchronous detection of point mutations and MSI status in colorectal cancer
Source: Cancer Med. 2022 Dec 30;12(7):8367–77. doi: 10.1002/cam4.5557 (PMC10134345; doi:10.1002/cam4.5557)
Supplement: Supplementary file 7 — Table S7. [file CAM4-12-8367-s003.doc]

**Supplementary Table 7** Patient test results and comparison of results from different test methods.

| **Patient** | **MASE-CE/Point mutation detection results** | **MASE-CE/MSI** | **NGS/Point mutation detection results** | **PCR-CE/MSI** | **Whether the results of the MASE-CE method were consistent with the results of the NGS and PCR-CE methods? Y(consistent), N(inconsistent)** |
| --- | --- | --- | --- | --- | --- |
| P1 | / | MSS | / | MSS | Y |
| P2 | / | MSS | / | MSS | Y |
| P3 | KRAS A146P | MSS | KRAS p.A146P 16.35% PIK3CA p.M1043I 19.98% APC p.T1556Kfs*10 3.77% SMAD4 p.R361C 23.79% | MSS | Y |
| P4 | KRAS G12D | MSS | KRAS G12D | MSS | Y |
| P5 | KRAS G12V | MSS | KRAS G12V | MSS | Y |
| P6 | KRAS G12A | MSS | KRAS G12A | MSS | Y |
| P7 | KRAS G13D | MSS | KRAS G13D | MSS | Y |
| P8 | / | MSS | / | MSS | Y |
| P9 | KRAS G12V | MSS | KRAS p.G12V 25.00% APC p.R1450* 26.63% | MSS | Y |
| P10 | / | MSS | / | MSS | Y |
| P11 | / | MSS | / | MSS | Y |
| P12 | / | MSS | / | MSS | Y |
| P13 | / | MSS | / | MSS | Y |
| P14 | KRAS G13D | MSI-L(B25) | KRAS G13D | MSS | N |
| P15 | / | MSI-H(D2/D17) | / | MSI-H(D2/D17) | Y |
| P16 | / | MSS | / | MSS | Y |
| P17 | / | MSS | / | MSS | Y |
| P18 | KRAS G12C | MSS | KRAS G12C | MSS | Y |
| P19 | / | MSS | / | MSS | Y |
| P20 | / | MSS | KRAS p.A59delA 24.30% APC p.S1465Wfs*3 9.99% | MSS | Y |
| P21 | KRAS G12V | MSS | KRAS G12V | MSS | Y |
| P22 | / | MSS | / | MSS | Y |
| P23 | KRAS G12R | MSS | KRAS G12R | MSS | Y |
| P24 | KRAS G12R | MSS | KRAS G12R | MSS | Y |
| P25 | / | MSS | KRAS p.V14I 17.19% APC p.R1450* 20.43% GNAS p.R199C 14.86% | MSS | Y |
| P26 | BRAF V600E | MSS | BRAF V600E | MSS | Y |
| P27 | KRAS G12C | MSS | KRAS G12C | MSS | Y |
| P28 | KRAS G12V | MSS | KRAS G12V | MSS | Y |
| P29 | / | MSS | NRAS p.Q61H 40.68% | MSS | Y |
| P30 | KRAS G12S | MSS | KRAS G12S | MSS | Y |
| P31 | / | MSS | / | MSS | Y |
| P32 | / | MSS | / | MSS | Y |
| P33 | KRAS G12D | MSS | KRAS G12D | MSS | Y |
| P34 | KRAS G12V | MSS | KRAS G12V | MSS | Y |
| P35 | / | MSS | / | MSS | Y |
| P36 | / | MSS | / | MSS | Y |
| P37 | / | MSS | / | MSS | Y |
| P38 | KRAS G12D | MSS | KRAS G12D | MSS | Y |
| P39 | / | MSS | / | MSS | Y |
| P40 | KRAS G13D | MSS | KRAS G13D | MSS | Y |
| P41 | / | MSS | / | MSS | Y |
| P42 | / | MSS | / | MSS | Y |
| P43 | / | MSS | / | MSS | Y |
| P44 | / | MSS | NRAS p.Q61L 12.90% ERBB2 p.V842I 18.68% | MSS | Y |
| P45 | KRAS G12D | MSS | KRAS G12D | MSS | Y |
| P46 | KRAS G13D | MSS | KRAS G13D | MSS | Y |
| P47 | KRAS G12D | MSS | KRAS G12D | MSS | Y |
| P48 | KRAS G13D | MSS | KRAS G13D | MSS | Y |
| P49 | KRAS G12S | MSS | KRAS G12S | MSS | Y |
| P50 | KRAS G12S | MSS | KRAS G12S | MSS | Y |
| P51 | / | MSS | / | MSS | Y |
| P52 | KRAS G12R | MSS | KRAS G12R | MSS | Y |
| P53 | / | MSS | / | MSS | Y |
| P54 | KRAS G12D | MSS | KRAS G12D | MSS | Y |
| P55 | KRAS A146P | MSS | KRAS A146P | MSS | Y |
| P56 | / | MSS | / | MSS | Y |
| P57 | / | MSS | / | MSS | Y |
| P58 | KRAS A146T | MSS | KRAS A146T | MSS | Y |
| P59 | / | MSS | / | MSS | Y |
| P60 | / | MSI-H(B25/B26) | / | MSI-H(B25/B26) | Y |
| P61 | / | MSI-H(B25/B26/D2/D17) | / | MSI-H（B25/B26/D2) | Y |
| P62 | / | MSS | / | MSS | Y |
| P63 | / | MSS | / | MSS | Y |
| P64 | KRAS G13D | MSI-H(B25/B26/D2) | KRAS G13D | MSI-H(B25/B26) | Y |
| P65 | / | MSI-L(D5) | / | MSS | N |
| P66 | / | MSS | / | MSS | Y |
| P67 | KRAS A146T | MSS | KRAS A146T | MSS | Y |
| P68 | KRAS G13D | MSI-H(B25/B26/D5) | KRAS G13D | MSI-H(B25/B26/D5) | Y |
| P69 | / | MSS | / | MSS | Y |
| P70 | / | MSS | / | MSS | Y |
| P71 | KRAS G13D | MSS | KRAS G13D | MSS | Y |
| P72 | KRAS G12D | MSS | KRAS G12D | MSS | Y |
| P73 | / | MSS | / | MSS | Y |
| P74 | / | MSS | / | MSS | Y |
| P75 | / | MSS | / | MSS | Y |
| P76 | KRAS G12V | MSS | KRAS G12V | MSS | Y |
| P77 | KRAS G12D | MSS | KRAS G12D | MSS | Y |
| P78 | / | MSS | / | MSS | Y |
| P79 | KRAS G12D | MSS | KRAS G12D | MSS | Y |
| P80 | KRAS G12D | MSS | KRAS G12D | MSS | Y |
| P81 | KRAS G13D | MSS | KRAS G13D | MSS | Y |
| P82 | / | MSS | / | MSS | Y |
| P83 | KRAS G12D | MSS | KRAS G12D | MSS | Y |
| P84 | / | MSS | / | MSS | Y |
| P85 | KRAS G12C | MSS | KRAS G12C | MSS | Y |
| P86 | KRAS G13D | MSS | KRAS G13D | MSS | Y |
| P87 | / | MSS | / | MSS | Y |
| P88 | KRAS G12C | MSS | KRAS G12C | MSS | Y |
| P89 | / | MSS | BRAF p.V600E 1.32% TP53 p.R175H 54.28% | MSS | N |
| P90 | / | MSS | / | MSS | Y |
| P91 | KRAS G13D | MSS | KRAS G13D | MSS | Y |
| P92 | KRAS G12S | MSS | KRAS G12S | MSS | Y |
| P93 | KRAS G12V | MSS | KRAS G12V | MSS | Y |
| P94 | / | MSS | KRAS p.G13C 26.44% TP53 p.M237I 49.26% | MSS | Y |
| P95 | / | MSS | / | MSS | Y |
| P96 | / | MSS | / | MSS | Y |
| P97 | / | MSS | / | MSS | Y |
| P98 | KRAS G12D | MSS | KRAS G12D | MSS | Y |
| P99 | KRAS G12D | MSS | KRAS G12D | MSS | Y |
| P100 | KRAS G12D | MSS | KRAS G12D | MSS | Y |
| P101 | KRAS A146T | MSS | KRAS A146T | MSS | Y |
| P102 | KRAS G13D | MSS | KRAS G13D | MSS | Y |
| P103 | / | MSS | / | MSS | Y |
| P104 | KRAS G12D | MSS | KRAS G12D | MSS | Y |
| P105 | / | MSS | / | MSS | Y |
| P106 | / | MSS | / | MSS | Y |
| P107 | NRAS G12D | MSI-H(B26/D5) | NRAS G12D | MSS | N |
| P108 | KRAS G13D | MSS | KRAS G13D | MSS | Y |
| P109 | KRAS G12V | MSS | KRAS G12V | MSS | Y |
| P110 | / | MSS | / | MSS | Y |
| P111 | / | MSS | KRAS p.G12D 35.95% APC p.R1450* 34.29% FBXW7 p.R425C 34.78% GNAS p.R201C 35.72% | MSS | N |
| P112 | KRAS G13D | MSS | KRAS G13D | MSS | Y |
| P113 | KRAS A146T | MSI-H(D5/D17) | KRAS A146T | MSS | N |
| P114 | KRAS G13D | MSS | KRAS G13D | MSS | Y |
| P115 | NRAS Q61K | MSS | NRAS Q61K | MSS | Y |
| P116 | / | MSS | / | MSS | Y |
| P117 | / | MSS | / | MSS | Y |
| P118 | / | MSS | / | MSS | Y |
| P119 | KRAS G12D | MSS | KRAS G12D | MSS | Y |
| P120 | / | MSS | / | MSS | Y |
| P121 | / | MSS | / | MSS | Y |
| P122 | KRAS G12C | MSS | KRAS G12C | MSS | Y |
| P123 | / | MSI-H(B26/D17) | / | MSI-H(B25/B26) | N |
| P124 | / | MSS | / | MSS | Y |
| P125 | KRAS Q61H | MSS | KRAS Q61H | MSS | Y |
| P126 | / | MSI-L(D2) | / | MSI-L(D2) | Y |
| P127 | / | MSI-L(D17) | / | MSI-L(D17) | Y |
| P128 | / | MSS | / | MSS | Y |
| P129 | KRAS G12D | MSS | KRAS G12D | MSS | Y |
| P130 | KRAS G12D | MSI-H(B25/B26/D5) | KRAS G12D | MSI-H(B25/B26/D5) | Y |
| P131 | KRAS G13D | MSS |  | MSS | N |
| P132 | KRAS A146T | MSS | KRAS A146T | MSS | Y |
| P133 | / | MSS | KRAS p.G13D 18.31% TP53 p.I195T 29.78% | MSS | N |
| P134 | KRAS G12D | MSS | KRAS G12D | MSS | Y |
| P135 | KRAS A146T | MSS | KRAS A146T | MSS | Y |
| P136 | KRAS G12D | MSI-L(B26) | KRAS G12D | MSI-H(B25/B26) | N |
| P137 | KRAS Q61H | MSS | KRAS Q61H | MSS | Y |
| P138 | / | MSS | / | MSS | Y |
| P139 | KRAS G12C | MSS | KRAS G12C | MSS | Y |
| P140 | KRAS G12V | MSS | KRAS G12V | MSS | Y |
| P141 | / | MSS | / | MSS | Y |
| P142 | / | MSS | / | MSS | Y |
| P143 | / | MSS | / | MSS | Y |
| P144 | / | MSS | / | MSS | Y |
| P145 | KRAS Q61L | MSS | KRAS Q61L | MSS | Y |
| P146 | / | MSS | / | MSS | Y |
| P147 | KRAS G12V | MSS | KRAS G12V | MSS | Y |
| P148 | KRAS G12V | MSS | KRAS G12V | MSS | Y |
| P149 | / | MSS | / | MSS | Y |
| P150 | KRAS G12V | MSS | KRAS G12V | MSS | Y |
| P151 | KRAS G12V | MSS | KRAS G12V | MSS | Y |
| P152 | / | MSS | / | MSS | Y |
| P153 | KRAS G13D | MSS | KRAS G13D | MSS | Y |
| P154 | KRAS G12V | MSS | KRAS G12V | MSS | Y |
| P155 | / | MSS | / | MSS | Y |
| P156 | / | MSS | / | MSS | Y |
| P157 | / | MSS | / | MSS | Y |
| P158 | / | MSS | / | MSS | Y |
| P159 | KRAS G12D | MSS | KRAS G12D | MSS | Y |
| P160 | / | MSS | / | MSS | Y |
| P161 | / | MSS | / | MSS | Y |
| P162 | / | MSS | / | MSS | Y |
| P163 | / | MSS | KRAS p.K117N 63.21% NRAS p.G13A 28.13% TP53 p.P151S 39.31% | MSS | Y |
| P164 | / | MSS | KRAS p.G12D 2.10% KRAS p.Q61H 32.89% | MSS | N |
| P165 | / | MSS | / | MSS | Y |
| P166 | BRAF V600E | MSS | KRAS p.V14I 1.08% PIK3CA p.H1047R 12.53% BRAF p.V600E 11.19% FBXW7 p.N598Y 11.80% FBXW7 p.R505C 2.73% TP53 p.P151R 9.90% | MSS | Y |
| P167 | KRAS G12V | MSS | KRAS G12V | MSS | Y |
| P168 | / | MSS | KRAS p.G12V 20.00% FBXW7 p.R505C 18.91% TP53 p.G245S 36.57% | MSS | Y |
| P169 | / | MSI-H(B25/B26/D2) | / | MSI-H(B25/B26) | N |
| P170 | / | MSS | / | MSS | Y |
| P171 | / | MSS | / | MSS | Y |
| P172 | KRAS G13D | MSI-H(B25/B26/D2) | KRAS G13D | MSI-H(B25/B26/D2/D5) | N |
| P173 | KRAS G12D | MSS | KRAS G12D | MSS | Y |
| P174 | / | MSS | / | MSS | Y |
| P175 | KRAS G13D | MSI-H(B25/B26/D2/D5) | KRAS G13D | MSI-H(B25/B26/D2/D5) | Y |
| P176 | KRAS G13D | MSI-H(B25/B26) | KRAS G13D | MSI-H(B25/B26) | Y |
| P177 | / | MSS | / | MSS | Y |
| P178 | KRAS G12D | MSS | KRAS G12D | MSS | Y |
| P179 | / | MSS | / | MSS | Y |
| P180 | / | MSS | / | MSS | Y |
| P181 | / | MSS | / | MSS | Y |
| P182 | KRAS Q61H | MSS | KRAS Q61H | MSS | Y |
| P183 | KRAS G12D | MSS | KRAS G12D | MSS | Y |
| P184 | KRAS G12V | MSS | KRAS G12V | MSS | Y |
| P185 | / | MSS | / | MSS | Y |
| P186 | KRAS G13D | MSS | KRAS G13D | MSS | Y |
| P187 | / | MSI-H(D2/D5/D17) | / | MSS | N |
| P188 | KRAS G12D | MSS | KRAS G12D | MSS | Y |
| P189 | / | MSS | / | MSS | Y |
| P190 | KRAS G12D | MSS | KRAS G12D | MSS | Y |
