# Supplementary material for: An innovative single‐base extension method for synchronous detection of point mutations and MSI status in colorectal cancer
Source: Cancer Med. 2022 Dec 30;12(7):8367–77. doi: 10.1002/cam4.5557 (PMC10134345; doi:10.1002/cam4.5557)
Supplement: Supplementary file 8 — Table S8. [file CAM4-12-8367-s008.doc]

**Supplementary Table 8** Predictive values of MSI in detecting tumors between Capillary Electrophoresis and MASE-CE assay. Capillary Electrophoresis is regarded as a gold standard for detecting MSI.

| Microsatellite markers | Sensitivity† | Specificity‡ | PPV§ | NPV¶ |
| --- | --- | --- | --- | --- |
| MASE-CE assay | 91.7%  (11/12) | 98.3%  (175/178) | 78.6%  (11/14) | 99.4%  (175/176） |
| † Sensitivity = (MSI-H tumors / total number of MSI-H tumors) × 100%.  ‡ Specificity = (non-MSI-H tumors / total non-MSI-H tumors) × 100%.  §PPV = (MSI-H tumors / all MSI-H tumors) × 100%.  ¶ NPV = (non-MSI-H tumors /all non-MSI-H tumors) × 100%.  MSI-H: High microsatellite instability; NPV: Negative predictive value; PPV: Positive predictive value. | | | | |
