# Supplementary material for: An innovative single‐base extension method for synchronous detection of point mutations and MSI status in colorectal cancer
Source: Cancer Med. 2022 Dec 30;12(7):8367–77. doi: 10.1002/cam4.5557 (PMC10134345; doi:10.1002/cam4.5557)
Supplement: Supplementary file 9 — Table S9. [file CAM4-12-8367-s006.doc]

**Supplementary Table 9**. Predictive values of KRAS/NRAS/BRAF mutations in detecting tumors between NGS and MASE-CE assay.

| KRAS/NRAS/BRAF | Sensitivity† | Specificity‡ | PPV§ | NPV¶ |
| --- | --- | --- | --- | --- |
| MASE-CE assay | 95.4%  (62/65) | 100.0%  (125/125) | 100.0%  (62/62) | 97.6%  (125/128) |
| † Sensitivity = TP / (TP + FN) × 100%.  ‡ Specificity = TN / (FP+TN) × 100%.  §PPV = TP / (TP + FP) × 100%.  ¶ NPV = TN / (FN + TN) x 100%.  TP: Ture positive; FP: False Positive; FN: False Negative: TN: True Negative. | | | | |
